# A cross-sectional study to identify the determinants of non-communicable diseases among fishermen in Southern India

**DOI:** 10.1186/s12889-021-10376-w

**Published:** 2021-02-27

**Authors:** Akhila Doddamani, A. B. Kirthinath Ballala, Sharath P. Madhyastha, Asha Kamath, Muralidhar M. Kulkarni

**Affiliations:** 1 Department of Community Medicine, Kasturba Medical College, Manipal Academy of Higher Education, Manipal, Karnataka 576104 India; 2 Department of General Medicine, Kasturba Medical College, Manipal Academy of Higher Education, Manipal, Karnataka 576104 India; 3 Department of Data Science, Prasanna School of Public Health, Manipal Academy of Higher Education, Manipal, Karnataka 576104 India

**Keywords:** NCDs, Fishermen, Lifestyle factors, Substance use, SDG 3

## Abstract

**Background:**

India is currently facing a rising epidemic of Non-Communicable Diseases (NCDs). Identification of modifiable risk factors is of paramount importance to curb this menace. Fishermen are one of the most vulnerable occupational groups with unique characteristics that make them prone to acquire NCDs, as a significant share of their life is spent at sea. Hence, this study was planned to ascertain the burden of NCDs, determine various risk factors of NCDs, and measure the association between risk factors and NCDs among fishermen of Coastal Karnataka in South India.

**Methods:**

A cross-sectional study was conducted among 681 fishermen aged 18 years and above as per the semi-structured interview schedule for two years (2017–2019). A convenience sampling strategy was adopted. The data was entered and analyzed using SPSS v.15.0. The results were described in terms of proportions and their 95% confidence intervals. Continuous data were summarized using the mean and standard deviation or median and interquartile range depending on the skewness of data. Chi-square test was used to study the association between NCDs and modifiable risk factors. Multiple logistic regression was used to identify risk factors of NCDs.

**Results:**

The mean (SD) age of the population was 42.5 (SD 12.5) years. The mean years involved in fishing was 19.8 years (SD 10.9). More than half (59.5%) of the study participants had severe stress and most (80.3%) were ever substance users. Advancing age, not being able to contact family while at sea, poor dietary practice, ever substance use, increasing waist circumference were significant correlates of NCDs.

**Conclusions:**

The commonly prevalent risk factors of NCDs among fishermen included poor dietary practice, higher stress levels, substance use, increasing waist circumference, and inability to contact with family while at sea. Hypertension and Diabetes were the two common NCDs in the study population. There is a need for immediate attention in managing NCDs’ risk factors by promoting a healthy lifestyle by primary health care providers through a sustainable community awareness program targeting fishermen at a convenient time and location, either at the sea-port or meeting places. Harmful effects of substance use, healthy dietary practices, and the importance of physical activity outside their job need emphasis. In addition, screening programs should be organized with the help of boat owners and fishing associations at-least once a year to pick up NCDs at an early stage.

**Supplementary Information:**

The online version contains supplementary material available at 10.1186/s12889-021-10376-w.

## Background

Every year approximately three-fourths of all deaths are due to Non-Communicable Diseases (NCDs) and amount to 41 million people globally, of which 15 million die between the ages of 30–69 years, and a majority of these “premature” deaths occur in low and middle-income countries [1]. The NCD burden has increased over the last decade resulting in an obstacle to achieving sustainable development goals (SDG) of poverty decline, financial stability, human security, and health equity [2]. The demographic and epidemiological transition happening in India is affecting the health of the people [3]. NCDs are becoming the most significant threat to health and development while still combating infectious diseases (like tuberculosis) and maternal and child health-related problems [4]. India alone contributes to more than two-thirds of the total NCD deaths in the South-East Asia Region (SEAR), with 5.87 million (60%) of all deaths in the country [5]. NCD management interventions are essential for achieving the global target of a 25% relative reduction in the risk of premature mortality from NCDs by 2025 and the SDG 3 target of a one-third reduction in premature deaths from NCDs by 2030 [1].

Tobacco consumption, physical inactivity, inappropriate diet, and alcohol abuse are the four behavioural risk factors for major NCDs. These risk factors are responsible for high blood glucose, elevated blood pressure, and raised BMI (body mass index). There are several determinants for these risk factors like social, financial, professional, cultural, etc., which are rooted in the surroundings and are undergoing quick transition [4]. There is a need for intervention strategies at primary health care with increasing chronic disease rates related to lifestyle behavioural risk factors.

Globally, India ranks third in fisheries and is an ancient and one of the essential livelihood options of the inhabitants of the coastal line of the country since the time immemorial [6]. Fishermen constitute the most disadvantaged group of people as they bear the rejection of health care reforms in terms of receiving these services [7]. Undoubtedly, fishing is a stressful and hazardous occupation that presents challenging physical conditions, separation, displacements with less than ideal personal habits [8–13]. Every launch into the ocean is a question of survival for the fishermen making them prone to multiple lifestyle diseases [14].

Although the literature is available on this economically important occupational group, it is limited to few risk factors of NCDs. In order to reduce the NCD burden, there is a need to comprehensively understand the profile of risk factors of NCDs, which in turn will help to develop appropriate intervention strategies. Hence this study was planned to ascertain the magnitude of NCDs and determine the various modifiable risk factors of NCDs among fishermen and measure the association between risk factors and NCDs.

## Methods

### Study design and sampling

A cross-sectional study was carried out to identify the determinants of NCDs among the fishermen aged 18 years and above in Coastal Karnataka in collaboration with Fishermen’s Association at the local Port on their scheduled holidays of 1st and 5th of every month during working hours. If it was not possible to meet them at the stipulated time and date, they were contacted at their houses after prior intimation. This study was conducted over two years (2017–2019).

The total number of registered boats in the fishing harbor was 837, with 15–25 fishermen in each boat. The number of fishermen registered and working at the fishing harbor was approximately 12,500. The types of boat included country boat (motorized or non-motorized that does not venture into the deep sea and returns to shore in 24 h with a duration of work ranging from 6 to 15 h depending on the catch of fish and season); deep-sea trawling boat-short duration (motorized, mechanized boats which venture into the deep sea and returns to shore within 24 h with a duration of work ranging between 18 and 22 h) and deep-sea trawling boat-long duration (motorized, mechanized boats which venture into the deep sea and return to shore ranging over 3–10 days). Using a convenient sampling method, 681 fishermen were included in the study.

### Sample size

According to the study done in a neighboring state [15], the least prevalence of NCDs’ risk factors among fishermen was smoking, which was reported to be 20.7%. Thus, sample size for a relative precision of 15% and a confidence interval of 95% worked out to be 681. We considered a non-response rate of 10% leading to a total sample of 750.

### Ethical consideration

The study was approved by the Kasturba Medical College and Kasturba Hospital Institutional Ethics Committee before the study’s initiation (IEC 575/2017). Permission from the President of the fishermen association and written informed consent from each fisherman were obtained before recruiting participants. The study was prospectively registered in the Clinical Trials Registry of India- CTRI/2017/12/010731. The data obtained during the study were kept confidential and anonymized before analysis.

### Data collection and measurements

The data were collected by personal interviews using a pre-designed and pretested interview schedule (Supplementary file). After explaining the purpose of the visit using a participant information sheet, written informed consent was obtained in local language from each eligible participant before recruiting them into the study.

The questionnaire comprised of socio-demographic characteristics, lifestyle factors, treatment history of chronic conditions, anthropometric measurements, and general physical examination, which was pilot tested and refined for collecting data. Socioeconomic Scale (SES) was ascertained using modified BG Prasad Scale 2018 [16] wherein Class 1 is ≥ Rs. 6574 (upper class), Class 2 is Rs. 3287–6573 (upper-middle class), Class 3 is Rs.1972–3286 (middle class), Class 4 is Rs.986–1971 (lower middle class), and Class 5 is ≤ Rs.985 (lower class). Random blood sugar was tested for all participants, while the lipid profile [17] was estimated in a sub-sample of 154 fishermen that constituted 20% of the total sample size. The description of variables and other operational definitions are elaborated in Table 1.

**Table 1:** Description of variables and operational definitions used in the study

Variables		
**Dietary habits**: According to study done by Kini S et al [18], type of diet whether vegetarian or a mixed diet was noted. The food items were grouped as follows:		
Red meat Fatty food Salty food	Mutton [19], pork and beef Butter and fried items (e.g-fried fishes) Pickle, papad, dried fish, chips (locally available)	
Energy rich foods Protective foods	Egg [20–22] and Fish [23] Green leafy vegetables, fruits	
The frequency of these food items was asked in last 1 month. By operational definition; frequency of intake of *unhealthy food items* were categorized as:		
Daily or 4–7 times per week 2 -3 times per week Occasionally/less frequently than once a week	score 1 score 3 score 5	
Not consuming the item	score 7	
Frequency of intake of *healthy food* were categorized similarly and were reverse scored.		
Information about consumption of extra salt (above the table) in the meals and usage of saturated oil (coconut oil, palm oil) while cooking was also taken.		
Yes No	Score 1 Score 7	
Overall, the scores were summed up and categorized into tertiles as Poor dietary practice (Score 0-33), Satisfactory dietary practice (Score 34- 38) and Good dietary practice (Score>38) for analysis		
**Sleep Scale from the Medical Outcomes Study:** [24] A 12-item screening tool was used to assess quality of sleep. Each negatively worded item (3 and 5 to 11) were rated on a 3-point likert scale with responses 1 to 3 with 3 points for good score, 2 for average and 1 for poor. Positively worded items 4 and 12 were reverse scored in 3-point likert scale. Item 1 was rated on a 5-point likert scale where in response 1 was scored as 3 (good), 2 and 3 were merged and was scored as 2(Average) and response 4 and 5 were scored as 1(Poor). Item 2 was an open-ended question wherein hours of sleep each night in last 1 week was asked and response of 7-8 hours of sleep was scored as 3 and anything < 7 hours or > 8 hours was scored as 1(poor). Subsequently, mean of all 12 items were taken and classified into 3 categories:		
Poor Average Good	Score 1-1.67 Score 1.67-2.34 Score 2.34-3	
**Substance use :**[25]	**Never-substance user:** A person who had never used any tobacco (smoking/ smokeless form) and alcohol anytime in life. **Ever-Substance user:** A person who has consumed either tobacco (smoking / smokeless form) or alcohol, currently or anytime in the past. **Current substance user:** Consuming any kind of the substance (tobacco and/or alcohol) in past one year. **Past substance user:** Quit tobacco or alcohol consumption for more than one year and has not used since then.	
**Tobacco and Alcohol use:**	These were defined in line with substance use [25]	
**Lipid Profile:**	**Unfavourable:** If anyone or all of the parameter/s is deranged. **Favourable:** If all the parameters are within the specified range. Biological reference interval of parameters: (As per ATP III guidelines) [17] 1. Total Cholesterol-140-200mg/dl; 2. Triglycerides-60-150mg/dl 3. HDL Cholesterol-40-60mg/dl 4. LDL Cholesterol-50-130 mg/dl	

### Diagnosis of NCDs

Hypertension: A known case and is on regular medication or participants on examination found to be having blood pressure > 140/90 mmHg as per JNC VIII criteria [26].

Diabetes mellitus: A known case and is on regular medication or participants found to have RBS > 200 mg/dl along with a sign or symptom of diabetes mellitus like polyuria, polyphagia, polydipsia, unexplained weight loss > 10% body weight, tingling sensation, or numbness in limbs, sudden vision changes as per ADA guidelines [27].

Cancer: A self-reported case of any cancer by the participants.

### Assessment of risk factors

Physical activity was measured as per Ramachandran et al. physical activity scale [28]. Stress was measured as per Perceived Stress Scale 4 [29]. Height, weight, and waist circumference were measured using standard precautions [30, 31]. BMI and waist circumference were classified as per WHO criteria [31, 32]. Lipid profile was assessed by drawing a venous blood sample of 3 ml using aseptic precautions and was classified as per ATP III guidelines [17, 33–35]. Data collection on other risk factors is elaborated in Table 1.

### Statistical methods

The collected data were entered and analyzed using SPSS software (Statistical Package for Social Sciences) V.26.0 (IBM SPSS, Bangalore) for windows. A subset of the sample was cross-checked for data entry by a co-investigator. The results were described in terms of proportions and their 95% confidence intervals. Continuous data were summarized using the mean and standard deviation or median and interquartile range depending on the skewness of data. Chi-square test was used to study the association, and *p*-value < 0.05 was considered statistically significant. Multivariable logistic regression was used to identify significant risk factors of NCDs, considering the covariates significant in the bivariate analysis with a p-value less than or equal to 0.2.

## Results

### Socio-demographic and lifestyle characteristics

Among the 750 fishermen who participated in the study, 681 (90.8%) consented and participated in the study. Among the 681 fishermen who participated in the study, missing data were not seen in any variables. The mean age of the participants in the study was 42.5 years (SD 12.5). Among the 681 fishermen, 55.5% were in the age group of 30–49 years. Almost three-fourth (74.4%) of the study participants were ever married, and most (97.1%) belonged to the Hindu religion. More than half (68.7%) of the study subjects had 5–10 years of schooling, and 45.7% of the study participants lived in a nuclear family. One third (32.7%) of the study subjects belonged to Class III according to modified BG Prasad Socio-economic classification 2018 [16]. Study participants were predominantly using country boats for their fishing activity (47.6%). More than half of the fishermen (57.6%) spent above 10 h in the sea, and around 38.8% of participants worked at shallow depths. 41.1% of subjects had spent more than 20 years in fishing activity and the mean years involved in fishing was 19.8 years (SD 10.85) in the current study. The study participants who could not contact the family at the shore while fishing was 42.6%. This finding almost corresponds with the type of fishing activity; as farther they ventured into the sea like the deep-sea trawlers, they could not contact the family at shore. Other details about socio-demographic and occupational characteristics are depicted in Table 2.

**Table 2:** Association of modifiable and non-modifiable risk factors with NCDs (*n* = 681)

Characteristics	NCDs					
Variables	Present(***N*** = 171)	Absent(***N*** = 510)	Crude OR (95% CI)	***p*** value	Adjusted OR* (95% CI)	p value^**#**^
	n (%)	n (%)				
**Age category (in years)**						
18–29	6 (3.5)	104 (20.4)	1	< 0.001	1	< 0.001
30–39	19 (11.1)	171 (33.5)	1.9 (0.8–5.0)		2.0 (0.7–5.4)	
40–49	82 (48.0)	106 (20.8)	13.4 (5.6–32.1)		11.9 (4.7–30.3)	
50–59	34 (19.9)	87 (17.1)	6.8 (2.7–16.9)		4.6 (1.7–12.2)	
≥ 60	30 (17.5)	42 (8.2)	12.4 (4.8–31.9)		9.0 (3.2–25.6)	
**Socio Economic Class (As per modified BG Prasad scale)**						
1 (upper)	29 (17.0)	50 (9.8)	1	0.01	1	0.02
2 (upper middle)	447 (27.5)	147 (28.8)	0.6 (0.3–1.0)		0.6 (0.3–1.2)	
3 (middle)	41 (24.0)	182 (35.7)	0.4 (0.2–0.7)		0.3 (0.2–0.7)	
4(lower middle)	47 (27.5)	111 (21.8)	0.73 (0.4–1.3)		0.7 (0.4–1.5)	
5 (lower)	7 (4.1)	20 (3.9)	0.6 (0.2–1.6)		0.6 (0.2–2.0)	
**Marital status**						
Never married	17 (9.9)	87 (17.1)	1	0.03	1	0.51
Ever married	154 (90.1)	423 (82.9)	1.9 (1.1–3.2)		1.3 (0.6–2.4)	
**Religion**						
Hindu	166 (97.1)	495 (97.1)	1	0.16	1	0.696
Others	5 (2.9)	15 (2.9)	1.0 (0.4–2.8)		0.8 (0.2–2.6)	
**Literacy status (years of schooling)**						
< 5	28 (16.4)	85 (16.6)	1	0.19	1	0.5
5–10	125 (73.1)	343 (67. 3)	1.1 (0.7–1.8)		1.3 (0.8–2.3)	
> 10	18 (10.5)	82 (16.1)	0.7 (0.3–1.3)		0.95 (0.4–2.0)	
**Type of family**						
Nuclear	77 (45.0)	234 (45.9)	1	0.87		NS
Joint	69 (40.4)	210 (41.2)	1.0 (0.7–1.5)		–	
Three generation	25 (14.6)	66 (12.9)	1.2 (0.7–2.0)		–	
**Type of fishing activity**						
Country boat	82 (48.0)	242 (47.4)	1	0.84		NS
Deep sea trawling boat- Short duration	50 (29.2)	160 (31.4)	0.9 (0.6–1.4)		–	
Deep sea trawling boat- Long duration	39 (22.8)	108 (21.2)	1.1 (0.7–1.7)		–	
**Years of fishing**						
< 5	8 (4.7)	71 (13.9)	1	< 0.001		
6–10	8 (4.7)	72 (14.1)	1.0 (0.4–2.8)			
11–15	26 (15.2)	85 (16.7)	2.7 (1.2–6.4)			
16–20	40 (23.4)	91 (17.8)	3.9 (1.7–8.9)			
> 20	89 (52.0)	191 (37.5)	4.1 (1.9–9.0)			
**Contact with family at shore while fishing**						
Yes	72 (42.1)	301 (59.0)	1	< 0.001	1	0.001
No	99 (57.9)	209 (41.0)	2.0 (1.4–2.8)		2.0 (1.3–3.0)	
**Dietary practice**						
Good	38 (22.2)	176 (34.5)	1	0.002	1	0.045
Satisfactory	50 (29.3)	156 (30.6)	1.5 (1.0–2.4)		1.5 (0.9–2.5)	
Poor	83 (48.5)	178 (34.9)	2.2 (1.4–3.3)		1.9 (1.2–3.2)	
**Physical activity**						
Sedentary	8 (4.7)	22 (4.3)	1	0.46		NS
Moderate	151 (88.3)	464 (91.0)	0.9 (0.4–2.1)		–	
Heavy	12 (7.0)	24 (4.7)	1.4 (0.5–4.0)		–	
**Stress levels**						
Mild	11 (6.4)	56 (11.0)	1	0.01	1	0.15
Moderate	42 (24.6)	167 (32.7)	1.3 (0.6–2.7)		1.6 (0.7–3.9)	
Severe	118 (69.0)	287 (56.3)	2.1 (1.1–4.1)		2.1 (0.9–4.8)	
**Sleep quality**						
Average	105 (61.4)	323 (63.3)	1	0.25		NS
Poor	16 (9.4)	29 (5.7)	1.7 (0.9–3.2)		–	
Good	50 (29.2)	158 (31.0)	0.97 (0.7–1.4)		–	
**Any substance use in life**						
Never	12 (7.0)	84 (16.5)	1	0.002	1	0.01
Ever	159 (93.0)	426 (83.5)	2.61 (1.4–4.9)		2.5 (1.2–5.2)	
**BMI**						
Normal + underweight	69 (40.4%)	224 (43.9)	1	0.67		
Overweight	77 (45.0)	221 (43.3)	1.1 (0.8–1.7)			
Obese	25 (14.6)	65 (12.8)	1.3 (0.7–2.1)			
**Waist Circumference (in cm)**						
Healthy (< 94)	55 (32.2)	335 (65.7)	1	< 0.001	1	< 0.001
Increased risk (94–102)	88 (51.4)	164 (32.2)	3.3 (2.2–4.8)		2.2 (1.4–3.4)	
Greatly increased risk (> 102)	28 (16.4)	11 (2.1)	15.5 (7.3–32.9)		8.4 (3.6–19.7)	
**Lipid profile status (*n*** **= 154)**						
Favourable	5 (12.8)	26 (22.6)	1	0.2		NS
Unfavourable	34 (87.2)	89 (77.4)	2.0 (0.7–5.6)		–	

*mutually adjusted for all the variables which has p < 0.2 for Crude OR

^#^*p* < 0.05 is considered to be statistically significant

#### NCDs among fishermen

**Fig. 1:**
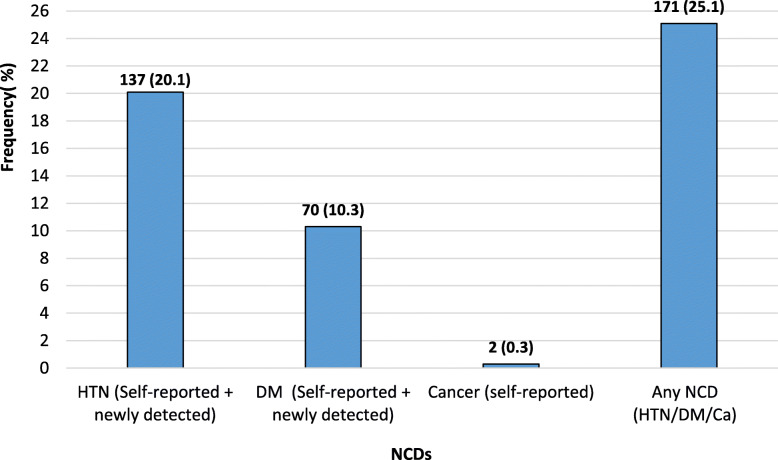
Prevalence of NCDs among study participants (*n* = 681)

Among the 681 study participants, hypertension (HTN) was present in 137 (20.1%), Diabetes Mellitus (DM) in 70(10.3%) and cancer (Ca) in 2(0.3%). Any NCD (HTN/DM/Ca) was present among 171(25.1%) of study subjects, as shown in Fig. 1.

#### Risk factors of NCDs among fishermen

**Fig. 2:**
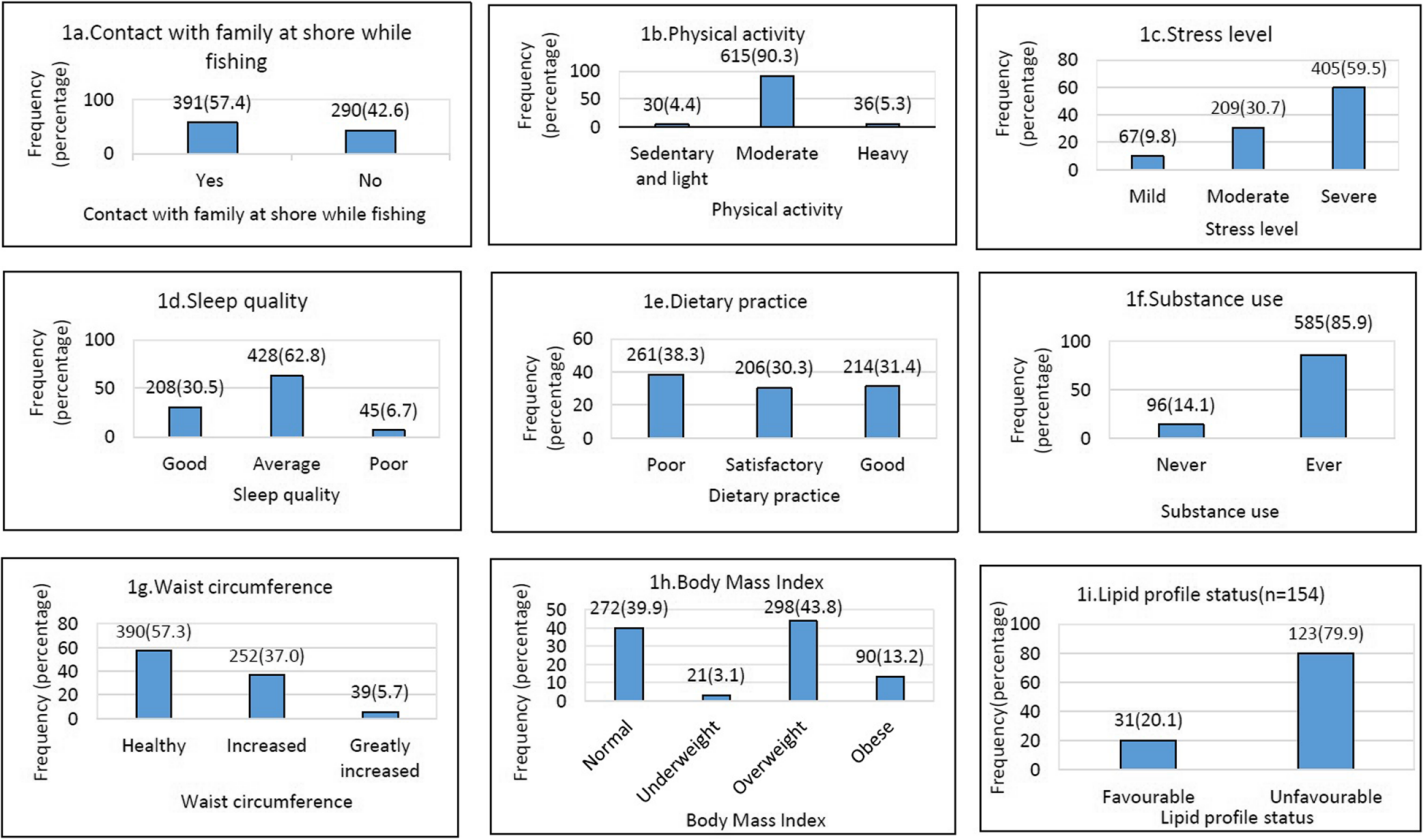
Graphical representation of modifiable risk factors of NCDs among fishermen (*n* = 681)

Among the total 681 fishermen, the reported risk factors of NCD included sedentary physical activity in 30(4.4%); severe stress in 405 (59.5%); average to poor sleep in 473 (69.5%); poor dietary practice in 261(38.3%), any substance use in 585(85.9%), increased waist circumference in 291 (42.7%) and higher BMI was observed among 388 (57%). Among the sub-sample tested for the lipid profile, 123 (79.9%) of 154 fishermen had unfavourable lipid profile status. A detailed depiction of risk factors of NCDs is shown in Fig. 2.

Any one of the NCD was present in 171 (25.1%) of the fishermen in the study and was significantly associated on bivariate analysis with advancing age group, lower socioeconomic status, ever married and increase in the number of years of fishing, contact with family at the shore while fishing, poor dietary practice, increase in stress levels, any substance use in life and increase in waist circumference(*p* < 0.05). Other factors like religion, type of family, literacy status, type of fishing activity, physical activity, sleep quality, BMI, and lipid profile status were not statistically significant. However, on multivariable logistic regression model after mutually adjusting with all the variables which had *p* < 0.2 in bivariate analysis and excluding years of fishing, fishermen with any NCD were more likely than those without any NCD to report having increasing waist circumference [AOR: (95% CI)-8.4(3.6–19.7)], any substance use [AOR: (95% CI)-2.5(1.2–5.2)], not being able to contact with family members while at sea [AOR: (95% CI)-2(1.3–3.0), poor dietary practice [AOR: (95% CI)-1.9(1.2–3.2)] and advancing age [AOR: (95% CI)-9.0(3.2–25.6)] as shown in Table 2. The age and duration of fishing being correlated, on replacing age with the duration of fishing in the multivariable analysis changes of AOR values were within 10%.

## Discussion

In this study, multivariable logistic regression showed that the risk factors like increasing waist circumference, any substance use in life, inability to contact with family while working at sea, poor dietary practice, advancing age, and lower socioeconomic status were significantly associated with NCDs.

There was a statistically significant association found between waist circumference and NCDs (*p* < 0.001). The odds of having NCDs in fishermen were 8.6 times higher in greatly increased risk and 2.2 times higher in increased risk than those with healthy waist circumference measurement. It was similar to the findings of Gopal M et al. [36]. However, BMI was not a statistically significant risk factor. In support of this finding, it has been documented in previous research that BMI does not distinguish between weight associated with muscle and weight associated with fat. As a result, the relationship between BMI and body fat content varies according to body build and proportion. It has also been shown previously that a given BMI may not correspond to the same degree of fatness across populations [37].

Ever use of a substance in life was identified as a risk factor in the study (*p* < 0.01). The odds of having NCDs were 2.5 times higher in participants with ever substance use than never substance use. This finding is in line with the study conducted by Gopal M et al. [36], wherein a significant association was found between duration of alcohol use and prevalence of hypertension. Likewise, a study by Kaerlev et al. [38] reported that Danish male seafarers faced an increased overall cancer risk of 1.26 (95% CI 1.19–1.32) times higher, especially for lung and tobacco-related cancers and alcohol-associated cancers. Although global evidence suggests that substance use is a significant risk factor to NCDs, tobacco and alcohol use, continue to be common practices among fishermen. This emphasizes regular structured substance use cessation programs at workplaces to bring about desired behaviour change and reduce the uptake of these habits.

Voyage to the sea itself is a stress factor for fishermen as the life at sea is unpredictable due to the sea winds and tides that can topple the boat anytime and result in drowning. Every launch into the ocean is a question of survival for the fishermen. Fishermen who could not contact family while venturing into the deep sea were significantly associated with NCDs (*p* < 0.001). Not being able to contact the family is a risk factor that could cause stress to the fishermen regarding their safety while fishing and the family’s safety and health at the shore and lead to NCDs. This factor was hypothesized in previous research [39], but our study provides evidence for the same. There is a need to develop cost-effective measures to enable communication between fishermen and their families even while in the deep sea and thus alleviate their stress and mitigate the effect due to this risk factor.

The poor dietary practice was an important risk factor for NCDs in the current study. The odds of having NCDs among fishermen with poor dietary practice was 1.9 times higher than those with good dietary practice. Our findings are similar to the study by Lawrie et al. [14], wherein significant association was found with poor dietary practices like less consumption of fruit and vegetables, higher consumption of fried food and snacks with high-fat content, and erratic meal timings at sea. This risk factor needs to be focused on, and appropriate interventions like dietary diversity can encourage fishermen to follow a healthy diet [2]. There is also a need to suggest a suitable modality to practice dietary diversity as it could be challenging for fishermen to store vegetables and fruits while they set off for fishing for days together.

Advancing age was found to be significantly associated with NCDs among fishermen (*p* < 0.001) as universally observed due to biological reasons. The odds of study subjects having NCDs were 11.4 times higher in the 40–49 years than 18–29 years age group. It was similar to the study done by Mudgal SM et al. [23] and Gopal M et al. [36]. Although increasing age is an important determinant for HTN, the age group in which maximum risk of HTN was observed was 40–49 years in our study compared to the age group above 55 years in the study by Gopal M et al. [36]. The probable reason for this difference could be because the study was carried out among fishermen currently involved in fishing activity, and elderly fishermen not involved presently in fishing activity owing to health conditions arising out of NCDs were not included in the study. These findings suggest that screening for NCDs should begin at an early age of fishermen and be integrated with sustainable health awareness programs at a convenient time and location.

The overall prevalence of any one of the NCDs (HTN/DM/Cancer) among fishermen in this study was 25.1%, which is higher than that in Kerala’s fishing community [15], wherein the prevalence of any chronic disease was 18.7%. This variation could be due to the study done among the fishing community and not on fishermen exclusively who venture into the sea as in the present study. However, our study’s primary objective was to find out about risk factors and not all NCDs.

Our study findings suggest that modifiable risk factors of NCD are commonly present among fishermen. A focussed approach to encourage them to adopt healthy dietary practices, adequate physical activity, relaxation techniques [39], and abstinence from substance use is essential. There is also a need for further research in designing novel interventions for the primary prevention of NCDs among challenging to reach occupational groups to achieve the SDG 3 target of reducing one-third of premature mortality from NCDs by 2030.

### Strengths

In general, data on the prevalence of modifiable risk factors for NCDs among fishermen is minimal. To our knowledge, this is the first study that has been carried out among fishermen in a developing country for assessing the risk factors of NCDs comprehensively. Also, to the best of our knowledge, no studies have explored the association of not having contact with family at the shore while fishing with NCDs, which could be a potential cause of stress. This study also provides important baseline data on the prevalence of modifiable risk factors of NCDs among fishermen for inclusion in follow-up studies of a longitudinal design.

### Limitations

This study has certain limitations. Sampling bias could have occurred in this study as convenience sampling strategy was adopted, leading to under-estimation of NCD risk factors. As we have excluded fishermen who are not actively involved in fishing activity in the last year, prevalence bias may have occurred, and we may have underestimated NCDs in our study. These might be the fishermen who may have NCDs and have retired from fishing due to the same reason. However, our primary objective was to find about risk factors that wouldn’t have been affected by this methodology. Generalizability is limited to the fishermen who are actively involved in fishing and living in similar geographical areas. Interviewer bias was minimal since a single interviewer conducted all the interviews using a standardized interview guide. We don’t anticipate recall bias to impact the findings since long-term aspects significantly and minute details of risk factors were not measured. As mentioned in the methodology section, standard methods and guidelines were followed during various measurements to prevent measurement bias. Lipid profile could not be done for the entire study population due to financial constraints. It could have led to a lower proportion of hyperlipidemia in the study population. As a result, it might not be depicted as a significant risk factor of NCD in this study. Cancer prevalence may be low in this study, as it was only self-reported.

## Conclusion

NCDs were found in one-fourth of the study population. Increasing waist circumference, substance use in life, inability to contact with family while at sea, poor dietary practice, advancing age, and low socioeconomic status were significantly associated with NCDs. Novel applications could be utilized to ensure that fishermen have contact with family at the shore while fishing. Fishermen could also benefit from dietary diversity; however, the modality has to be thought of to store vegetables while they set off for fishing for days together. Although time and again, research has revealed that modifiable risk factors of NCDs are prevalent and are associated with NCDs among fishermen, no concrete actions have been taken among this vulnerable population. To achieve SDG 3 by 2030, there is a dire need for a focused approach to alleviating the risk factors of NCDs among fishermen. Hence there is a need for immediate attention in terms of management of risk factors of NCDs by the promotion of healthy lifestyle by primary health care providers through sustainable community awareness program targeting fishermen at a convenient time and location, either at the sea-port or their meeting places. Harmful effects of substance use, healthy dietary practices, and the importance of physical activity outside their job must be emphasized. In addition, screening programs should be organized with the help of boat owners and fishing associations at-least once a year to pick up NCDs at an early stage. Policymakers and stakeholders in the health sector need to institute population-based strategies to create awareness about these select NCD risk factors and their consequences. Further longitudinal studies should be undertaken to generate representative data on NCD risk factors and inform appropriate interventions for reducing the NCD burden among people involved in fishing and other at-risk populations.

## Supplementary Information

**Additional file 1.**

## Data Availability

The datasets used and analyzed during the current study are available from the corresponding author on reasonable request.

## Abbreviations

BMI: Body mass index
BP: Blood pressure,
Ca: Cancer
CI: Confidence interval
CVD: Cardiovascular disease
DM: Diabetes Mellitus
GRBS: General random blood sugar
HDL: High-density lipoproteins
HTN: Hypertension
IEC: Institutional Ethics Committee
IQR: Interquartile range
JNC 8: Joint national Committee 8
KMC: Kasturba Medical College
LDL: Low-density lipoproteins
NCD: Non-communicable disease
OR: Odds ratio
SD: Standard deviation
SHR: Standardized hospitalization ratios
SIR: Standardized incidence ratios
SPSS: Statistical Package for the Social Sciences
WHO: World Health Organization
