# Supplementary material for: A cross-sectional study to identify the determinants of non-communicable diseases among fishermen in Southern India
Source: BMC Public Health. 2021 Feb 27;21:414. doi: 10.1186/s12889-021-10376-w (PMC7913403; doi:10.1186/s12889-021-10376-w)
Supplement: Supplementary file 1 — Additional file 1. [file 12889_2021_10376_MOESM1_ESM.docx]

**A cross-sectional study to identify the determinants of**

**Non-communicable diseases among Fishermen in Southern India**

**Individual Proforma**

ID no: Date:

**I. Socio Demographic Details:**

**1. Age: ______________** years

| Hindu | Muslim | Christian | Others(specify) |
| --- | --- | --- | --- |

**2. Religion:**

| Married | Unmarried | Separated | Widowed | Divorced |
| --- | --- | --- | --- | --- |

**3. Marital status:**

| Illiterate | Primary school  (Ist to 4th std) | Middle school  (5^th^ to 7^th^ std) | Secondary school  (8th to 10th std) | Higher secondary  (11^th^ -12^th^ std) | Degree | Post graduate |
| --- | --- | --- | --- | --- | --- | --- |

**4. Literacy**

**Status**:

**5. Type of family:**

| Nuclear | 3 generation | Joint or extended |
| --- | --- | --- |

|  |
| --- |

**6. Number of members in the family:**

**7. Total income of the family per month (native) Rs.________________**

**8. Type of fishing activity:**

| Country boat | Deep sea trawlers-short duration | Deep sea trawlers-long duration |
| --- | --- | --- |

| Hrs |
| --- |

**9. Average time spent for fishing in a day:**

**10. No. of days involved in fishing in a week -----------------**

**11. No. of years involved in fishing: -------------**

| a. Yes **if yes how?** --------------------- |
| --- |
| b. No |

**12. Do you contact with family or people in the shore when at sea**

**II. Diet:**

1. If non vegetarian, mention whether you consume the following items:

| **Items** | **Yes** | **No** | **If yes, Frequency**  (daily/4-7 times per week-01) (2-3times/week=02)  (occasionally<once a week-03) |
| --- | --- | --- | --- |
| Egg |  |  |  |
| Fish |  |  |  |
| Chicken |  |  |  |
| Others  (Mutton, Pork, Beef) (Specify)----------------- |  |  |  |

2. Which **oil** do you use for cooking? Coconut oil/Palm oil/Pure ghee/Refined oil/any other (specify):--------------

3. Do you consume **extra salt** (other than used for cooking) in your daily meals? Yes/No

4. Is there a **difference** between what you consume at boat and home? Yes/No

5. Please indicate if you consume the following food items:

| **Food items** | **Yes** | **No** | **If Yes, frequency(daily/4-7 times per week-01)**  **(2-3times/week=02) (occasionally<once a week-03)** |
| --- | --- | --- | --- |
| Tea |  |  | (No. of times in a day) |
| Coffee |  |  | (No. of times in a day) |
| Butter |  |  |  |
| Fried items (Fried fish, sea food, etc.) |  |  |  |
| Pickles |  |  |  |
| Papad |  |  |  |
| Dried fish |  |  |  |
| Chips |  |  |  |
| Fruits |  |  |  |
| Green leafy Vegetables |  |  |  |
| Juice |  |  |  |

**III. Physical activity status:**

| **1** | Does your work involve mostly sitting/standing/walking? | **Yes** | |  | **Minimum score = 1** |
| --- | --- | --- | --- | --- | --- |
| **2** | Does your work involve moderate activity,  e.g. brisk walking, fetching water from wells, household work etc.? | **Duration/day** | **Score** | **Days/week**  **(multiply by number of days)** | **Maximum score** |
|  |  | **45 mins**  **>45mins-4 hrs**  **>4-8 hrs** | **2**  **3**  **4** |  | **28** |
| **3** | Does your work involve vigorous manual activity  (Occupation category 1)? | **15min-1hr**  **>1-4 hrs**  **>4-10hrs** | **5**  **6**  **7** |  | **49** |
| **4** | Additional activities:  Do you use a cycle or engage in  Sports activities? | **45 mins**  **>45mins-4 hrs**  **>4-8 hrs** | **1**  **2**  **3** |  | **21** |
|  | **TOTAL** | | | |  |

**Perceived Stress Scale 4:**

The questions in this scale ask you about your feelings and thoughts during **THE LAST MONTH.**

In each case, please indicate your response by placing an “X” over the square representing

**HOW OFTEN** you felt or thought a certain way:

|  | **During THE LAST MONTH, How often** | **Never**    **0** | **Almost**  **Never**  **1** | **Some**  **Times**  **2** | **Fairly**  **Often**  **3** | **Very**  **Often**  **4** |
| --- | --- | --- | --- | --- | --- | --- |
| **1.** | Have you felt that you were unable to control the Important things in your life? |  |  |  |  |  |
| **2.** | Have you felt confident about your ability to handle your Personal problems? |  |  |  |  |  |
| **3.** | Have you felt that things were going your way? |  |  |  |  |  |
| **4.** | Have you felt difficulties were piling up so high that you could not overcome them? |  |  |  |  |  |

**Total score: -----------------------------**

**VI. Sleep Scale**

**1. How long did it usually take for you to fall asleep during the past 1week?**

- 0-15 minutes…………..……….1
- 16-30 minutes………………….2
- 31-45 minutes………………….3
- 46-60 minutes………………….4
- More than 60 minutes …….5

**2. On the average, how many hours did you sleep each night during the past 1 Week?**

Number of hours per night: --------------------

**0=None of the Time 1=A Little of the Time 2= Some of the Time 3=A Good Bit of the Time 4= Most of the Time 5= All of the Time**

| **How often during the past 1 week did you…** | **0** | **1** | **2** | **3** | **4** | **5** |
| --- | --- | --- | --- | --- | --- | --- |
| 3.Feel that your sleep was not quiet  (Moving restlessly, feeling tense, Speaking, etc. while sleeping)? |  |  |  |  |  |  |
| 4. Get enough sleep to feel rested upon waking in the morning? |  |  |  |  |  |  |
| 5. Awaken short of breath or with a headache? |  |  |  |  |  |  |
| 6. Feel drowsy or sleepy during the day? |  |  |  |  |  |  |
| 7. Have trouble falling asleep? |  |  |  |  |  |  |
| 8. Awaken during your sleep time and have trouble falling asleep again? |  |  |  |  |  |  |
| 9. Have trouble staying awake during the day? |  |  |  |  |  |  |
| 10. Snore during your sleep? |  |  |  |  |  |  |
| 11. Take naps (5 minutes or longer) during the day? |  |  |  |  |  |  |
| 12. Get the amount of sleep you needed? |  |  |  |  |  |  |

**Score: ………………**

**VII. HABITS:**

**Any substance abuse:**

| Tobacco Smoking | Tobacco Chewing | Snuff | Drinking | Others(specify) |
| --- | --- | --- | --- | --- |

**Vii a. Tobacco use:**

| Yes | No |
| --- | --- |

| **Currently** | **Past**(stopped 1 year ago and not using now) |
| --- | --- |

If yes,

**VII b) ALCOHOL USE:**

| A.**YES** | 1. **Currently** |
| --- | --- |
|  | 1. **Past**   (stopped one year ago and not using now at all) |
| B. **NO** | |

1) Have you ever consumed any form of alcohol?

**HYPERTENSION:**

a. Hypertension status: Yes/No/Don’t know

b. Are you on treatment for hypertension? Yes/ No

If yes,

| Salt restricted diet From how long? ------------- | Drugs From how long? -------------- | Any other (mention) -------------- From how long? -------------- |
| --- | --- | --- |

**DIABETES MELLITUS:**

a. Diabetes status: Yes /No/ don’t know

b. Are you on treatment for diabetes? Yes/ No

If yes,

| Diabetic diet  From how long? ---------- | OHA Tablets(Oral Hypoglycaemic Agents )  From how long? ------------- | Insulin injection  From how long? --------- | Both OHA tablets and Insulin |
| --- | --- | --- | --- |

**Do you have history of any of the following (in the past 1 month) (tick-multiple answer possible)**

- Diabetes-Polyuria /Polydipsia /polyphagia /tingling numbness/weight loss -------------------------Yes/No
- Hypertension-Giddiness/headache/blurring of vision/swelling of legs/palpitations----------------Yes/No
- Heart disease- Dyspnoea on exertion / Shortness of breath----------------------------------------------Yes/No
- Any other (Specify) ______________________

**ANY CO-MORBIDITIES**:

|  | **Co-morbidity** | **Yes/No** | **Duration** | **Treatment taken** | | |
| --- | --- | --- | --- | --- | --- | --- |
|  |  |  |  | **Yes** | **No** | |
| 1. | Ischemic Heart disease / Coronary Artery Disease |  |  |  | |  |
| 2. | Stroke / Paralysis |  |  |  | |  |
| 2. | Hypercholesterolemia |  |  |  | |  |
| 3. | Asthma |  |  |  | |  |
| 4. | Liver diseases |  |  |  | |  |
| 5. | Chronic kidney Disease  (Renal failure/oliguria/polyuria) |  |  |  | |  |
| 6. | Any Cancer(Mention) |  |  |  | |  |
| 7. | Any other illness(mention) |  |  |  | |  |

A. Any time **admitted** for any illness? Yes/No

- If yes, reason for admission? --------------------------------------------------------------------------------
- How many days of admission? ------------------------------------------------------------------------------

B. Any illness/sickness **in past 15 days**? ----------------------------------------------------------------------

C. **Family history of** Diabetes Mellitus/Hypertension/Coronary artery disease/Stroke/Hypercholesterolemia/Liver disease/Kidney diseases/any cancer (Tick appropriate one).

Relation of the family member to the participant_________________________________

**XI. Examination:**

- Pulse rate: ----------------beats/min
- BP-1:--------------------------------------mm Hg , BP-2:----------mmHg(If BP 1> 140/90mm Hg)

**XII. Anthropometry:**

Height: …………………………………cm

Weight: ………………………………..kg

BMI : ………………………………..

Waist circumference: ……………cm

**XIII. Investigations:**

1. **GRBS:** __________mg/dl.
2. **Lipid profile**:
   - - Total Cholesterol --------------------mg /dl
     - Triglyceride: --------------------------- mg /dl
     - HDL: ---------------------------mg/dl
     - LDL: ---------------------------- mg/dl
     - TC/HDL ratio:------------------
